# Sequence variants in oxytocin pathway genes and preterm birth: a candidate gene association study

**DOI:** 10.1186/1471-2350-14-77

**Published:** 2013-07-26

**Authors:** Jinsil Kim, Kara J Stirling, Margaret E Cooper, Mario Ascoli, Allison M Momany, Erin L McDonald, Kelli K Ryckman, Lindsey Rhea, Kendra L Schaa, Viviana Cosentino, Enrique Gadow, Cesar Saleme, Min Shi, Mikko Hallman, Jevon Plunkett, Kari A Teramo, Louis J Muglia, Bjarke Feenstra, Frank Geller, Heather A Boyd, Mads Melbye, Mary L Marazita, John M Dagle, Jeffrey C Murray

**Affiliations:** 1 Department of Anatomy and Cell Biology, University of Iowa, Iowa City, IA 52242, USA; 2 Department of Pediatrics, University of Iowa, Iowa City, IA 52242, USA; 3 Center for Craniofacial and Dental Genetics, Department of Oral Biology, School of Dental Medicine, University of Pittsburgh, Pittsburgh, PA 15219, USA; 4 Department of Pharmacology, University of Iowa, Iowa City, IA 52242, USA; 5 Centro de Educación Médica e Inverstigaciones Clínicas, Buenos Aires 1431, Argentina; 6 Instituto de Maternidad y Ginecología Nuestra Señora de las Mercedes, San Miguel de Tucumán 4000, Argentina; 7 Biostatistics Branch, National Institute of Environmental Health Sciences, Research Triangle Park, NC 27709, USA; 8 Institute of Clinical Medicine, Department of Pediatrics, University of Oulu, Oulu 90014, Finland; 9 Human and Statistical Genetics Program, Washington University, St. Louis, MO 63110, USA; 10 Department of Obstetrics and Gynecology, University of Helsinki, Helsinki 00290, Finland; 11 Perinatal Institute, Cincinnati Children's Hospital Medical Center and Department of Pediatrics, University of Cincinnati College of Medicine, Cincinnati, OH 45229, USA; 12 Department of Epidemiology Research, Statens Serum Institut, Copenhagen, S 2300, Denmark; 13 Department of Human Genetics, Graduate School of Public Health, University of Pittsburgh, Pittsburgh, PA 15219, USA

**Keywords:** Preterm birth, Genetic association analysis, Oxytocin pathway, Single nucleotide polymorphism, Rare variant

## Abstract

**Background:**

Preterm birth (PTB) is a complex disorder associated with significant neonatal mortality and morbidity and long-term adverse health consequences. Multiple lines of evidence suggest that genetic factors play an important role in its etiology. This study was designed to identify genetic variation associated with PTB in oxytocin pathway genes whose role in parturition is well known.

**Methods:**

To identify common genetic variants predisposing to PTB, we genotyped 16 single nucleotide polymorphisms (SNPs) in the oxytocin (*OXT*), oxytocin receptor (*OXTR*), and leucyl/cystinyl aminopeptidase (*LNPEP*) genes in 651 case infants from the U.S. and one or both of their parents. In addition, we examined the role of rare genetic variation in susceptibility to PTB by conducting direct sequence analysis of *OXTR* in 1394 cases and 1112 controls from the U.S., Argentina, Denmark, and Finland. This study was further extended to maternal triads (maternal grandparents-mother of a case infant, *N*=309). We also performed *in vitro* analysis of selected rare *OXTR* missense variants to evaluate their functional importance.

**Results:**

Maternal genetic effect analysis of the SNP genotype data revealed four SNPs in *LNPEP* that show significant association with prematurity. In our case–control sequence analysis, we detected fourteen coding variants in exon 3 of *OXTR*, all but four of which were found in cases only. Of the fourteen variants, three were previously unreported novel rare variants. When the sequence data from the maternal triads were analyzed using the transmission disequilibrium test, two common missense SNPs (rs4686302 and rs237902) in *OXTR* showed suggestive association for three gestational age subgroups. *In vitro* functional assays showed a significant difference in ligand binding between wild-type and two mutant receptors.

**Conclusions:**

Our study suggests an association between maternal common polymorphisms in *LNPEP* and susceptibility to PTB. Maternal *OXTR* missense SNPs rs4686302 and rs237902 may have gestational age-dependent effects on prematurity. Most of the *OXTR* rare variants identified do not appear to significantly contribute to the risk of PTB, but those shown to affect receptor function in our *in vitro* study warrant further investigation. Future studies with larger sample sizes are needed to confirm the findings of this study.

## Background

Preterm birth (PTB) is a major public health problem that accounts for approximately 10% of all births worldwide [1,2]. In 2011, PTBs, occurring before 37 completed weeks of gestation, comprised 11.73% of live births in the United States [3]. Prematurity is a leading cause of perinatal mortality and in survivors is a significant contributor to short- and long-term morbidity [4]. The etiology of PTB is not completely understood, but multiple lines of evidence suggest that genetic factors play an important role in PTB, including a high rate of recurrence in individuals with a history of previous preterm delivery (PTD), a tendency to occur within families, and racial disparity [1,5-7].

The oxytocin (OXT)-oxytocin receptor (OXTR) system provides promising candidate genes for studies of genetic contributions to prematurity. Its components influence a wide range of physiological, behavioral, and emotional processes in humans, but have been most extensively studied for their role in reproduction, particularly labor and parturition [8,9]. OXT acts as an inducer of uterine contraction and the myometrium becomes increasingly sensitive to the action of OXT towards term [8]. This increase in uterine sensitivity to OXT occurs concomitantly with an upregulation of *OXTR* mRNA and a dramatic increase in myometrial OXTR number, which peaks during early labor [8,9]. The OXTR is a G protein-coupled receptor, which, upon stimulation, activates various intracellular signaling pathways such as the phosphoinositide cascade, eventually leading to uterine contraction [8,10,11]. The availability of OXT to the receptor is dependent on serum levels of leucyl/cystinyl aminopeptidase (LNPEP), an enzyme that hydrolyzes and inactivates the hormone [12,13]. LNPEP, also known as PLAP, increases in maternal serum during pregnancy and plays an important role in fetal development as well as pregnancy maintenance via regulating OXT levels and activity [12,13]. The efficacy of Atosiban, an OXT antagonist used to stop premature uterine contractions and delay PTD [14], provides further support for the importance of this hormonal system in prematurity.

Previous candidate gene association studies have provided evidence for genetic variation predisposing to PTB with four maternal and one fetal variants showing relatively consistent evidence of association [15]. In the present study, we hypothesize that allelic variations in *OXT*, *OXTR*, and *LNPEP* contribute to the genetic predisposition to prematurity. We evaluated the association between maternal and fetal genotypes of single nucleotide polymorphisms (SNPs) in the three genes and PTD in U.S. patients. We also performed direct sequence analysis in four population groups to search for rare variants in the OXT-OXTR system (particularly in the *OXTR* gene) that might play a role in PTB. Selected rare *OXTR* variants identified in preterm cases were then further investigated to determine their functional significance.

## Methods

### Study population

The study population consisted of case and control patients from 4 countries, including the U.S. (4 sites; the University of Iowa Hospitals and Clinics in Iowa City, IA, Magee-Womens Hospital in Pittsburgh, PA, University of Rochester Medical Center in Rochester, NY, and Wake Forest University in Wake Forest, NC), Argentina (two centers; Instituto de Maternidad y Ginecología Nuestra Señora de las Mercedes in Tucumán and Hospital Provincial de Rosario in Rosario), Finland (the University of Helsinki in Helsinki), and Denmark (the island of Funen and the Danish National Birth Cohort (DNBC) [16]). All families provided signed informed consent for study enrollment in accordance with the protocols approved by research ethics committees in the U.S. (the University of Iowa Institutional Review Board (IRB), University of Pittsburgh IRB, University of Rochester Research Subjects Review Board, Wake Forest University Health Sciences IRB), Argentina (the Research Ethics Committee of Centro de Educación Médica e Investigaciones Clínicas), Finland (the Ethics Committee of the University Central Hospital, Helsinki), and Denmark (the Scientific-Ethical Committee of the Southern Danish Region and the Biomedical Research Ethics Committee of the Capital City Region of Denmark). PTD was defined as delivery before 37 completed weeks of gestation. Gestational age (GA) was determined by obstetrical assignment using the first day of the last menstrual period as well as ultrasound examination, and was confirmed by assessment at birth. Demographic and clinical data were collected by chart review and/or clinician interview. Table 1 summarizes demographic characteristics of each population studied. U.S. cases were families with either a spontaneous or medically indicated preterm birth - of note, twin births were excluded at the data analysis step. Cases from Finland (mother-infant dyads, <36 weeks’ gestation) were families with spontaneous onset of preterm singleton birth from 2003 to 2009, selected using the following exclusion criteria: elective deliveries without spontaneous onset of labor and deliveries in which either maternal (e.g. systemic infection) or fetal (e.g. malformation) disease with known predisposition to premature birth was indicated. Finnish control families were defined as those with two or more children, all of whom were delivered at 37 weeks or later. The median gestational ages for index mother-infant dyads were 34 and 40 weeks for cases and controls, respectively. Danish cases and controls were identified on Funen where the University Hospital of Odense was a major participating site for case collection, and from the DNBC [16]. The DNBC consisted of 96,946 total births, including 5,352 preterm births, occurring between 1997 and 2002. We selected 723 case (≤36 weeks gestation) and 920 control (40 weeks gestation) mothers for our study. Individuals were considered for inclusion in this study only if there was no evidence of obstetrical induction, placental abnormalities, pre-eclampsia, congenital malformations, and multiple births. Biological samples collected from mothers were stored in the Biobank at Statens Serum Institut in Copenhagen. Epidemiologic information was collected from existing medical records and from three maternal interviews, during the first trimester, at about 30 weeks gestation and 6 months after birth.

**Table 1:** Demographic characteristics of study populations

	**SNP genotyping**	**Resequencing**										
		**Case-control**^**3**^									**Maternal triad**^**4**^	
	**U.S.**	**Argentina**			**Denmark**			**U.S.**			**Argentina**	**U.S.**
	***N*=651**	**Case ( *N *=123)**	**Control ( *N *=63)**	***P***	**Case ( *N *=723)**	**Control ( *N *=920)**	***P***	**Case ( *N *=443)**	**Control ( *N *=34)**	***P***	***N*=187**	***N*=109**
**GA (weeks)**^**1**^	31.1 ± 3.7 (21–36)	33.1 ± 2.5 (27–36)	39.4 ± 0.9 (37–41)	*	33.7 ± 2.8 (20–36)	40	N/A	30.3 ± 3.8 (22–36)	38.9 ± 1.1 (37–41)	*	32.6 ± 3.0 (23–36)	32.0 ± 3.8 (24–36)
											[Unknown: 5]	
**Infant BW (grams)**^**1**^	1739.4 ± 814.7 (332–4610)	1834.8 ± 514 (830–3200)	3514.1 ± 487 (2430–4950)	*	2444 ± 648 (250–5270)	3720.7 ± 457 (2400–5250)	*	1606 ± 791 (332–4400)	3495.2 ± 417.9 (2380–4275)	*	1763.3 ± 527.2 (615–3040)	1967.5 ± 778.2 (597–3691)
											[Unknown: 5]	
**Infant gender (male/female)**^**2**^	373/278	65/57	31/32	0.64	385/338	482/438	0.77	249/194	20/14	0.85	99/87	54/55
											[Unknown: 1]	
**Maternal age at delivery (years)**^**1**^	27.8 ± 6.2 (14–46)	24.7 ± 6.5 (15–48)	26.0 ± 6.1 (16–40)	0.22	29.1 ± 4.4 (17–44)	29.7 ± 4.0 (18–42)	0.001	27.7 ± 6.0 (14–44)	27.9 ± 5.3 (18–40)	0.86	23.2 ± 5.3 (16–39)	30.3 ± 5.2 (18–41)
	[Unknown: 15]										[Unknown: 3]	[Unknown: 11]

^1^Data are presented as mean ± standard deviation, with range in parenthesis.

^2^Data are presented as counts.

^3^Resequencing was also performed on Finnish cases (*N*=105) and controls (*N*=95). See the Methods section for a demographic description of this population.

^4^Data for Danish maternal triads (*N*=13) are not shown.

* <0.0001.

*GA:* gestational age, *BW:* birth weight, *N/A:* not available.

### SNP genotyping

A total of 812 premature infants born in Iowa between 1999 and 2008 with GAs between 21 and 36 weeks (mean, 31 ± 3.7 weeks) and one or both of their parents were selected for genotyping. DNA was extracted from cord blood or discarded venous blood from the infants and venous blood, buccal swab, or saliva from the parents using standard protocols. Genotyping for SNP markers was performed using TaqMan assays (Applied Biosystems, Foster City, CA, U.S.) as described in detail elsewhere [17]. A total of 16 tagging SNPs were selected to cover haplotype blocks of the oxytocin gene (*OXT*; 1 SNP), the oxytocin receptor gene (*OXTR*; 9 SNPs), and the leucyl/cystinyl aminopeptidase gene (*LNPEP*; 6 SNPs). The SNPs genotyped, along with their minor allele frequencies (MAFs), are listed in Table 2. Twin births were excluded, leaving 651 singleton infants for analysis. Of the 651 infants, 565 were successfully genotyped with GAs between 23 and 36 weeks (mean 31.1 ± 3.75).

**Table 2:** **List of SNPs in the *OXT, OXTR, *and *LNPEP *genes genotyped**

**Gene**	**SNP**	**Alleles**	**Chr**	**Position**^**1**^	**MAF**
*OXT*	rs2740210	G/T	20	3053255	0.32
*OXTR*	rs7632287	A/G	3	8791446	0.25
	rs11706648	A/C	3	8796547	0.33
	rs237887	A/G	3	8797042	0.41
	rs4686301	C/T	3	8798586	0.29
	rs237889	C/T	3	8802483	0.37
	rs53576	A/G	3	8804371	0.32
	rs237893	A/G	3	8805950	0.42
	rs237897	A/G	3	8808285	0.36
	rs4686302	C/T	3	8809222	0.12
*LNPEP*	rs4869315	A/G	5	96229272	0.44
	rs3849749	A/T	5	96234533	0.41
	rs4869317	A/T	5	96292004	0.28
	rs18059	C/T	5	96352068	0.48
	rs316206	C/T	5	96380733	0.32
	rs13175726	A/G	5	96387033	0.29

^1^Position according to NCBI Build 37.3 GRCh37.p5 assembly.

*SNP:* single nucleotide polymorphism, *Chr:* chromosome, *MAF:* minor allele frequency.

### Sequencing

A subgroup of the genotyped Iowa patients was selected for phase 1 sequencing based on GA, including 94 infants and 95 mothers of different infants with GA between 24–32 weeks. All three coding exons of *OXT* and all four exons of *OXTR* were sequenced in both directions, as described in prior studies [17]. Phase 2 (replication) sequencing, where we focused only on exon 3 of *OXTR*, was performed on cases and controls from Argentina (infants and mothers; case, *N*=212 and control, *N*=125), Denmark (mothers participating in the DNBC; case, *N*=723 and control, *N*=920), Finland (mothers; case, *N*=105 and control, *N*=95), and the U.S. (infants and mothers; case, *N*=719 and control, *N*=229). Removal of twin births as well as unsuccessfully genotyped individuals left a very small number of Argentinian and U.S. controls, making a case–control association analysis particularly for the Argentinian population impossible.

To assess the effect of maternal *OXTR* genotype on the risk of PTD, we performed sequence analysis on the same region of *OXTR* in maternal triads (each including a mother of a premature infant with GA between 23 and 36 weeks (single gestation, born after spontaneous onset of labor) and her parents) as well as in a small group of patients consisting of 71 case and 64 control mothers. These case and control mothers were all Caucasians from Iowa with a singleton spontaneous delivery between 2005 and 2010. Further exclusion criteria for cases include preeclampsia and induction of labor. Of the 71 cases (mothers of preterm infants with GA between 23–28 weeks and birth weight (BW) between 413 and 1770 grams) and the 64 controls (mothers of term infants with GA between 39–42 weeks and BW between 2050 and 4756 grams), 62 cases and 60 controls with complete data were included in the analysis. The maternal grandmother-grandfather-mother triads were created from 309 pedigrees from different populations, including Argentina (2 centers, *N*=187), Denmark (the island of Funen, *N*=13), and the U.S. (4 sites, *N*=109). The families were enrolled in the study between 2005 and 2010 (Argentina and U.S.) and between 2008 and 2009 (Denmark). Of note, the small sample size of the Danish group (*N*=13) made statistical analysis uninformative. DNA was extracted from venous blood or saliva (Argentina), saliva (Denmark), and venous blood, buccal swab, or saliva (U.S.) and subsequently used for sequencing. All sequencing was performed by Functional Biosciences, Inc., Madison, WI, U.S.

### Plasmids and cells

A full-length cDNA encoding human OXTR [GenBank: AY389507] was obtained from the Missouri S&T cDNA Resource Center (Rolla, MO, U.S.). Site-directed mutagenesis was performed to generate mutant constructs possessing missense mutations of potential functional significance identified in premature cases, including P108A, W203R, and F284. The mutations were introduced individually into the wild-type OXTR sequence using a PCR-based mutagenesis strategy. The wild-type and mutant full-length cDNAs were inserted into the EcoRI and XhoI sites of expression vector pcDNA3.1 (+) (Invitrogen, Carlsbad, CA, U.S.). The sequence of all constructs was confirmed by sequencing.

African green monkey kidney COS-7 cells were maintained in Dulbecco’s Modified Eagle’s Medium supplemented with 10% newborn calf serum, 50 μg/mL gentamicin, and 10 mM HEPES, pH 7.4. Cells were plated at a density of 4 x 10^5^ cells/well in 6-well (35-mm) plates, and transiently transfected with plasmid DNA using FuGENE® HD Transfection Reagent (Roche Applied Science, Indianapolis, IN, U.S.) according to the manufacturer’s instructions. After overnight incubation, the cells were washed and used in subsequent experiments.

### Inositol phosphate assays

The accumulation of inositol phosphates was measured using [^3^H]inositol-labeled cells incubated in the absence or presence of 100 nM oxytocin for 1 h as described elsewhere [18]. Results were presented as mean fold difference between stimulated and unstimulated values (in cpm/10^6^ cells) or mean ratio of the basal level in each transfection group to that in the wild-type group.

### Ligand binding assays

Two days after transfection, cells were washed twice with warm buffer (20 mM HEPES, 0.15 M NaCl, 1 mg/ml bovine serum albumin, pH 7.4), and incubated with 50 nM [^3^H]oxytocin (PerkinElmer, Waltham, MA, U.S.) alone (total binding) or together with 20 μM unlabeled oxytocin (nonspecific binding). After 2.5 hours of incubation at room temperature, the cells were washed twice with cold buffer and dissolved in 0.5 ml of 0.5 N NaOH overnight. After collecting the lysate, each well was washed with another 0.5 ml of 0.5 N NaOH, and the wash was combined with the lysate, followed by neutralization with 0.25 ml of 2 N HCl prior to measuring radioactivity. Specific binding was determined by calculating the difference between total and nonspecific binding and was expressed as mean cpm/10^6^ cells.

### Statistical analysis

The common SNP genotyping data were tested for genotype deviation from the Hardy–Weinberg equilibrium (HWE) using PLINK [19] and Haploview [20]. We first analyzed the data for all GAs together and then performed stratified analyses. For both maternal and fetal effects, five-week sliding windows of GA were utilized, starting with 21–25 weeks and ending with 32–36 weeks. For fetal effect analysis, three-stage stratification was additionally used, resulting in three GA groups: early (between 21 and 27 weeks), middle (between 28 and 30 weeks), and late (between 31 and 36 weeks). We performed a transmission disequilibrium test (TDT) using the Family Based Association Test (FBAT) software [21,22], which analyzes overtransmission of alleles to an affected fetus [23]. For maternal genetic effect analysis, we used a log-linear approach to study maternally-mediated effects [24]. This approach uses a case-parents triad as the analysis unit and it tests maternally-mediated genetic effects based on the symmetry assumption of allele counts between the mothers and the fathers in the source population [25]. We performed likelihood ratio tests of the maternal genetic effects. The expectation-maximization algorithm was applied to fully utilize families with missing parental genotypes [26]. We also performed a haplotype analysis for all the six SNPs in the *LNPEP* gene using TRIMM [27]. The test statistics was constructed based on the vector of genotype differences between the mother and the father. Under genetic mating symmetry assumption, the difference vector has an expected value of 0 at each locus under the null hypothesis. A permutation test was used to evaluate the significance of the test statistic.

The sequencing data from the maternal triads were analyzed in a similar way except that we defined as affected those (mothers) who delivered preterm infants and used GA and BW of the infants as phenotypes of the case mothers. We performed a TDT using the FBAT program [21,22] for each common or rare variant found in the sequenced region. The data were analyzed for all GAs together as well as for GA subgroups using five-week sliding windows of GA and three-stage (early, middle, and late) stratification as described above.

We performed chi-square and Fisher’s exact tests using SAS version 9.2 statistical program (SAS, Cary, NC, U.S.) for a 2 by 2 or 2 by 3 contingency table to analyze the sequence data on rare and common *OXTR* variants found in cases and controls from Argentina, Denmark, Finland, and the U.S. as well as a small group of Caucasian case and control mothers from Iowa. In the Iowa case and control study, we restricted our analysis to only 3 common polymorphisms (rs4686302, rs237902, and rs61740241) because of the absence of rare variant minor alleles in both cases and controls.

The Danish sequencing data were further analyzed using linear regression to model genetic effects of the *OXTR* variants identified on GA. In the Iowa case–control study, we conducted logistic regression analysis to predict case/control status and linear regression analyses to predict GA (weeks) and BW (grams) on the aforementioned three *OXTR* polymorphisms.

Data for inositol phosphate and ligand binding assays were analyzed using one-way ANOVA, followed by Dunnett's test with InStat software (GraphPad, La Jolla, CA, U.S.).

### Analysis of genetic variation in *OXTR* using publicly available reference data

The *OXTR* sequencing results from our study were additionally analyzed utilizing publicly available data from two databases: 1) the 1000 Genomes Project (1000GP) [28,29], which provides a catalogue of genetic variants present in the genomes of healthy individuals from different ethnic groups, and 2) the National Heart, Lung, and Blood Institute (NHLBI) Grand Opportunity (GO) Exome Sequencing Project (ESP) [30], which provides a catalogue of exome variants present in the genomes of approximately 2500 European and African American individuals from cohorts sampled for heart, lung, and blood diseases . No data on these for GA is reported. We investigated the two databases in search of coding variants in exon 3 of the *OXTR* gene in European Americans and African Americans, and then compared the variants retrieved with those identified in our sequencing study. The variants reported in the 1000GP (February 2012 release, 20120316) [28,29] were annotated using the SeattleSeq Annotation Server [31].

## Results

### Effects of common polymorphisms in the OXT pathway genes on risk of PTB

We selected 16 SNPs in the *OXT*, *OXTR*, and *LNPEP* genes based on haplotype block structures and examined them for their association with PTB in patients from the U.S. (Iowa). All SNPs tested had a MAF greater than 10% (Table 2). Excluding one SNP (rs237889) with HWE violation (*p*=8 x 10^-5^) and one SNP (rs4686302) with insufficient informative families (*n*<10) left a total of 14 SNPs for analysis.

#### Fetal genetic effects

**Figure 1:**
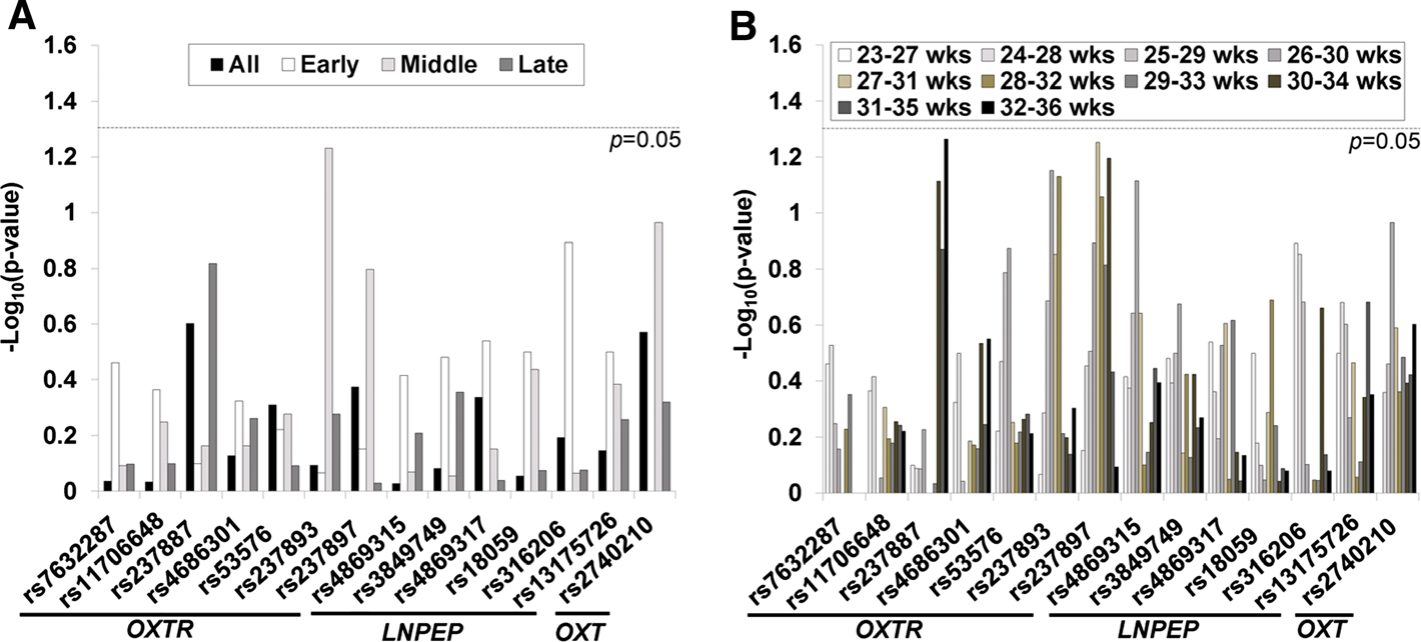
**Results of fetal genetic effect analysis.** No statistically significant (*p*<0.05) association was found between any of the SNPs in the *OXT*, *OXTR*, and *LNPEP* genes and preterm birth risk either when all GAs together were evaluated **(A)** or when three-stage **(A)** or five-week sliding window **(B)** stratification was used.

Figure 1 shows the *p*-values obtained from fetal effect analysis, using the entire set of data as well as subsets classified by GA. There was no significant SNP found either when all GAs were examined together (Figure 1A) or when the GA-stratified analyses were performed (Figure 1A and 1B).

#### Maternal genetic effects

**Figure 2:**
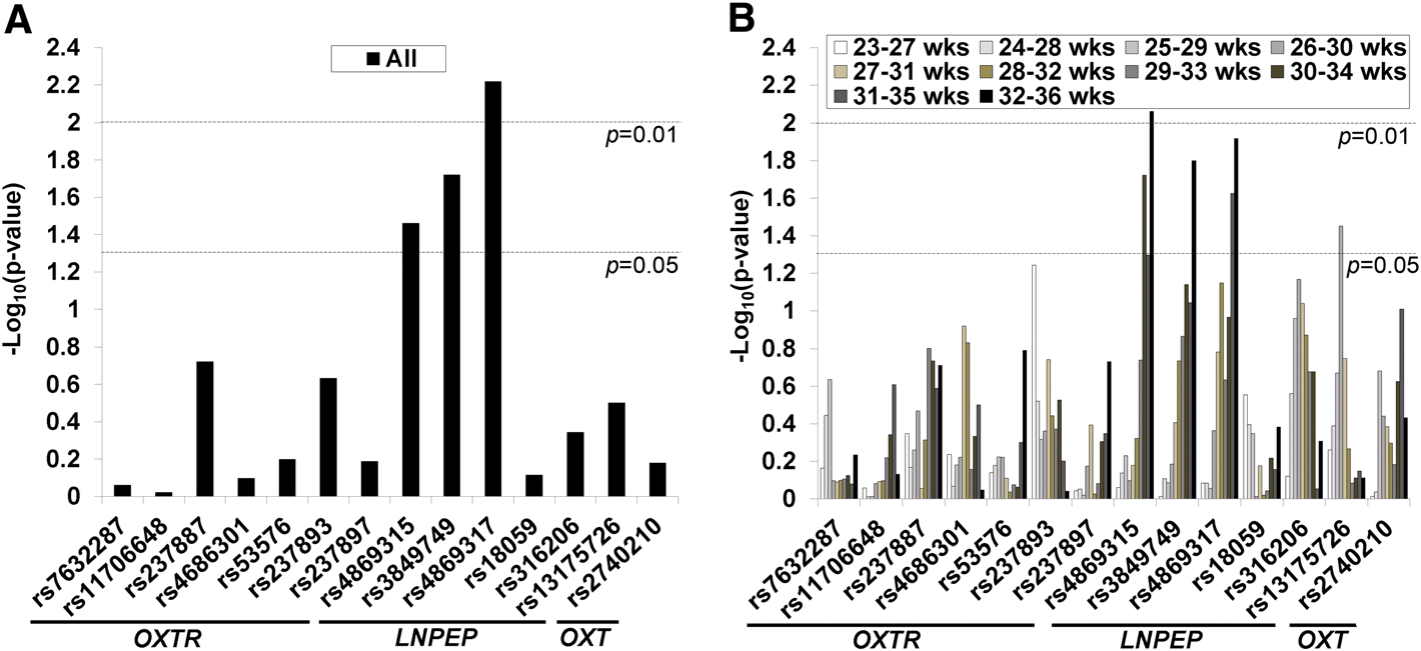
**Results of maternal genetic effect analysis.** Three SNPs (rs4869315, rs3849749, rs4869317) in the *LNPEP* gene were significantly associated with preterm birth when all GAs were together considered **(A)**. Another SNP (rs13175726) in *LNPEP* as well as the same three SNPs were found to be significant for several GA subgroups **(B)**.

Figure 2 summarizes the results from the analysis of maternally mediated genetic effects. When the analysis was performed on all GA cases (Figure 2A), three SNPs in *LNPEP* were found to have a *p*-value of less than 0.05 (rs4869315, *p*=0.035; rs3849749, *p*=0.019; rs4869317, *p*=0.006). These values are uncorrected for the multiple comparisons done in this study where a Bonferroni corrected *p*-value for significance would be <0.002 accounting for 14 SNPs and both maternal and fetal comparisons. When the analysis was stratified by GA using five-week sliding windows (Figure 2B), the same variants in *LNPEP* remained significant - rs4869315 for the 30–34 (*p*=0.019) and 32–36 (*p*=0.009) week GA groups, rs3849749 for the 32–36 (*p*=0.016) week GA group, and rs4869317 for the 31–35 (*p*=0.024) and 32–36 (*p*=0.012) week GA groups. Another *LNPEP* SNP, rs13175726 (*p*=0.035) was additionally found to be significant for the 26–30 week GA group. Haplotype analysis of all 6 SNPs in *LNPEP* failed to yield any significant results.

### Analysis of rare variants in the *OXTR* gene for the association with PTB

#### Rare sequence variations in OXTR: phase 1 and phase 2 case–control resequencing studies

Lack of association between common polymorphisms in *OXT* and *OXTR* led us to examine whether there exist(s) rare genetic variant(s) in these two genes with potential effects on the risk of prematurity. To this end, we first sequenced all exons of *OXT* and *OXTR* in a subgroup (*n*=189) of the genotyped (U.S.) patients. This pilot (hereinafter referred to as “phase 1”) sequencing analysis revealed only previously reported SNPs in the *OXT* gene. In *OXTR*, we found three rare variants (V172A, L206V, W203R, Table 3) that were not present in public databases at the time of the data analysis. Of note, the mutant allele at codon 206 was always detected together with that at codon 172, indicative of a rare double-mutant haplotype, while the V172A rare allele was identified both on its own and together with the L206V rare allele. We found that all the missense variants identified were located in one high-yield amplicon of *OXTR*, which corresponds to exon 3, and therefore attempted to replicate these phase 1 sequencing results in more individuals.

**Table 3:** **Summary of all *OXTR *coding variants detected by resequencing in the present study**

**Position**^**1**^	**Nucleotide variation**^**2**^	**Amino acid substitution**	**Domain location**	**dbSNP ID**	**Sequencing analysis**^**3**^	**Number of variant carriers**^**4**^			
						**Mother**		**Infant**	
						**Case**	**Control**	**Case**	**Control**
8809843	+31C>T	A11T	END		Case–control (P2)	1 (D)	0	0	0
8809741	+133C>A	V45L	TMD 1		Case–control (P2)	0	2 (D)	0	0
8809552	+322G>C	P108A	ECL 1	rs202138705	Case–control (P2), MT	1 (F)	0	0	0
8809446	+428T>C	Q143R	ICL 2		MT				
8809359	+515A>G	V172A	TMD 4	rs115324487	Case–control (P1 and P2), MT	7 (A, U)	0	5 (A, U)	0
8809267	+607A>G	W203R	ECL 2	rs200498154	Case–control (P1)	0	0	1 (U)	0
8809258	+616G>C	L206V^5^	TMD 5	rs150746704	Case–control (P1 and P2)	2 (U)	0	3 (A, U)	0
8809243	+631C>A	V211L	TMD 5		Case–control (P2)	1 (D)	1 (D)	0	0
8809222	+652C>T	A218T	TMD 5	rs4686302	All				
8809184	+690G>A	N230N	ICL 3	rs237902	All				
8809162	+712C>T	A238T	ICL 3	rs61740241	All				
8809150	+724C>G	E242Q	ICL 3		MT				
8809133	+741C>T	A247A	ICL 3		Case–control (P2)	2 (D)	1 (D)	0	0
8809119	+755C>G	G252A	ICL 3	rs151141371	Case–control (P2), MT	3 (D)	2 (D, F)	0	0
8809033	+841C>T	V281M	TMD 6	rs144814761	Case–control (P2)	1 (D)	0	0	0
8809024	+850A>G	F284L^6^	TMD 6	rs201783860	Case–control (P2)	1 (U)	0	0	0

^1^Position (on chromosome 3) according to NCBI Build 37.3 GRCh37.p5 assembly.

^2^Nucleotide numbering: +1 is the A of the ATG translation initiation codon.

^3^Sequencing analysis in which the variant indicated was detected. See text for further details.

^4^Counts of common variant (A218T, N230N, and A238T) carriers are not shown. Counts from maternal triad analysis were not included: P108A, Q143R, V172A, E242Q, and G252A were identified each in one family. Letter in parentheses is a population code: *A:* Argentina, *D:* Denmark, *F:* Finland, and *U:* U.S.

^5^Always found together with V172A.

^6^Identified in a twin case and not considered for formal association analysis.

*ECL:* extracellular loop, *END:* extracellular N-terminal domain, *ICL:* intracellular loop, *TMD:* transmembrane domain, *P1:* phase 1, *P2:* phase 2, *MT:* maternal triad.

**Figure 3:**
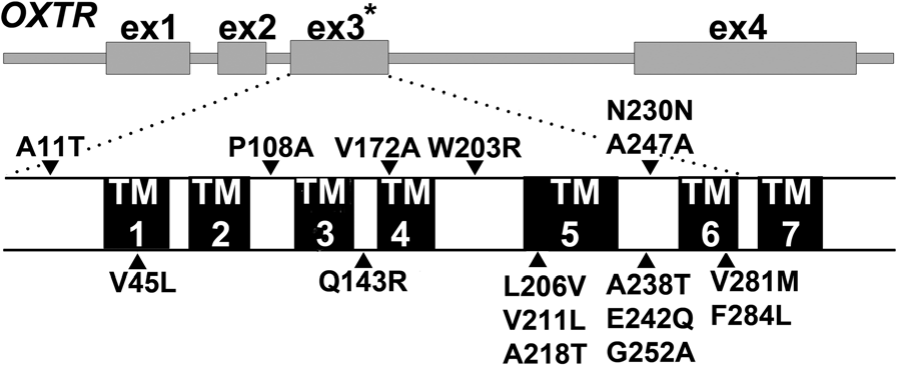
**Schematic representation of *OXTR *gene structure and location of all coding variants identified by sequencing.** Note that all the variants were found in exon 3 (indicated by an asterisk) that encodes 6 of 7 transmembrane domains of the receptor.

Phase 2 (replication) sequencing was performed on preterm cases and term controls from the U.S., Argentina, Finland, and Denmark (Table 1). The *OXTR* variants identified and their locations in the receptor are shown in Table 3 and Figure 3, respectively. The F284L mutation (rs201783860), which was detected in one case mother from the U.S., was also transmitted to the infant. In a well-characterized cohort of case (≤36 weeks gestation, *N*=723) and control (40 weeks gestation, *N*=920) mothers selected from the DNBC Study [16], we found three novel variants, including A11T, V211L, and A247A (Table 3). All but four (V45L, V211L, A247A, and G252A) rare coding variants identified in phase 1 and 2 studies were found in cases only.

To gain a more complete understanding of the spectrum of genetic variation in exon 3 of *OXTR* and the importance of the identified *OXTR* rare variants in predisposition to PTB, we examined recent data from the 1000 Genomes Project (1000GP) [28,29] and the NHLBI GO Exome Sequencing Project (NHLBI_ESP) [30] (see Methods for more details). A list of coding variants in exon 3 of *OXTR* reported in the two databases is shown in Additional file 1. We compared this list with our sequencing results and found 5 variants (V45L, V172A, L206V, G252A, and V281M) overlapping with our data in individuals of European origin. It was particularly noted that two (V172A and L206V) of the 5 variants occur at much higher frequencies in African Americans (MAF: V172A, 5.52% and L206V, 1.84%) than in European Americans (MAF: V172A, 0.01% and L206V, 0.01%). It would be interesting to explore if these variants play a role in prematurity, given that about 10% of normal individuals whose sequences are catalogued in the databases are expected to be preterm and that PTB rates are higher in African Americans than in whites [32]. In addition, we found 4 rare variants (A11T, P108A, W203R, and F284L) that are present exclusively in our cases with PTB. Importantly, three of them were predicted to have functional effects by PolyPhen [33] and SIFT [34] (Table 4), suggesting that they are potentially etiologic.

**Table 4:** ***In silico *prediction of functional significance of *OXTR *missense variants identified by resequencing**

**Amino acid change**	**PolyPhen**		**SIFT**	
	**Prediction**	**PSIC score difference**	**Prediction**	**Tolerance index**
A11T	Benign	N/A	Tolerated	0.44
V45L	Benign	N/A	Tolerated	0.11
P108A^*^	Probably damaging	2.243	Affect protein function	0.04
Q143R	Benign	0.286	Tolerated	0.67
V172A	Benign	0.820	Tolerated	0.37
W203R^*^	Probably damaging	4.115	Affect protein function	0.00
L206V	Benign	0.319	Tolerated	0.41
V211L	Benign	N/A	Tolerated	0.41
A218T	Benign	0.324	Tolerated	0.36
A238T	Benign	0.330	Tolerated	0.59
E242Q	Benign	0.740	Tolerated	0.59
G252A	Benign	0.278	Tolerated	0.74
V281M^*^	Probably damaging	2.013	Affect protein function	0.00
F284L^*^	Probably damaging	2.208	Affect protein function	0.01

^*^Amino acid substitutions with predicted deleterious effects.

Association analysis of all rare coding variants identified by phase 1 and 2 sequencing showed no significant results – either when the variants were tested individually or when all missense variants were combined and analyzed together (Table 5).

**Table 5:** **Association of *OXTR *rare coding variants with preterm birth in Danish, Finnish, and U.S. populations**

**Position**^**1**^	**Rare allele**	**Amino acid substitution**	**dbSNP ID**	**Population**			
				**Denmark**^**2**^	**Finland**^**2**^	**U.S.**^**3**^	
				***N*=723 (case) / 920 (control)**	***N*=105 (case) / 95 (control)**	***N*=443 (case) / 34 (control)**	
				**Mother**	**Mother**	**Mother**	**Infant**
8809843	T	A11T		0.43	-	-	-
8809741	A	V45L		0.51	-	-	-
8809552	C	P108A	rs202138705	-	1.00	-	-
8809359	G	V172A	rs115324487	-	-	1.00	1.00
8809267	G	W203R	rs200498154	-	-	N/A	N/A
8809258	C	L206V	rs150746704	-	-	1.00	1.00
8809243	A	V211L		1.00	-	-	-
8809133	T	A247A		1.00	-	-	-
8809119	G	G252A	rs151141371	0.33	0.43	-	-
8809033	T	V281M	rs144814761	0.44	-	-	-
All missense variants combined^**4**^				0.35	1.00	1.00	1.00

^1^Position (on chromosome 3) according to NCBI Build 37.3 GRCh37.p5 assembly.

^2^Subjects include mothers only.

^3^Subjects include both mothers and infants.

^4^All missense variants detected in each population were combined together for analysis.

- Variant not found in the population indicated.

We further extended our analysis on Danish mothers, in particular, and performed regression analyses to more precisely define the effects of *OXTR* genetic variation on GA. We modeled the effects of all coding variants (both common and rare) identified by sequencing on GA treated as either a continuous (in weeks) or categorical (preterm, ≤36 weeks; and term, 40 weeks) variable. However, no results were found to be significant in both analyses (Additional file 2).

#### Maternal OXTR sequence variation and susceptibility to PTB

To address in more depth the importance of maternal genetic effects on the risk of PTB, we extended the results obtained from the study of Danish mothers by performing sequence analysis of *OXTR* in maternal triads created from 309 pedigrees from the U.S., Argentina, and Denmark (Table 1) as well as in a small group of Caucasian patients from Iowa consisting of case (*n*=71) and control (*n*=64) mothers. Each maternal triad consisted of a mother of a preterm infant with GA between 23 and 36 weeks and her parents (grandmother-grandfather-mother), and we examined the transmission of *OXTR* variant alleles from maternal grandparents to the mothers of affected infants using the transmission disequilibrium test (TDT).

**Figure 4:**
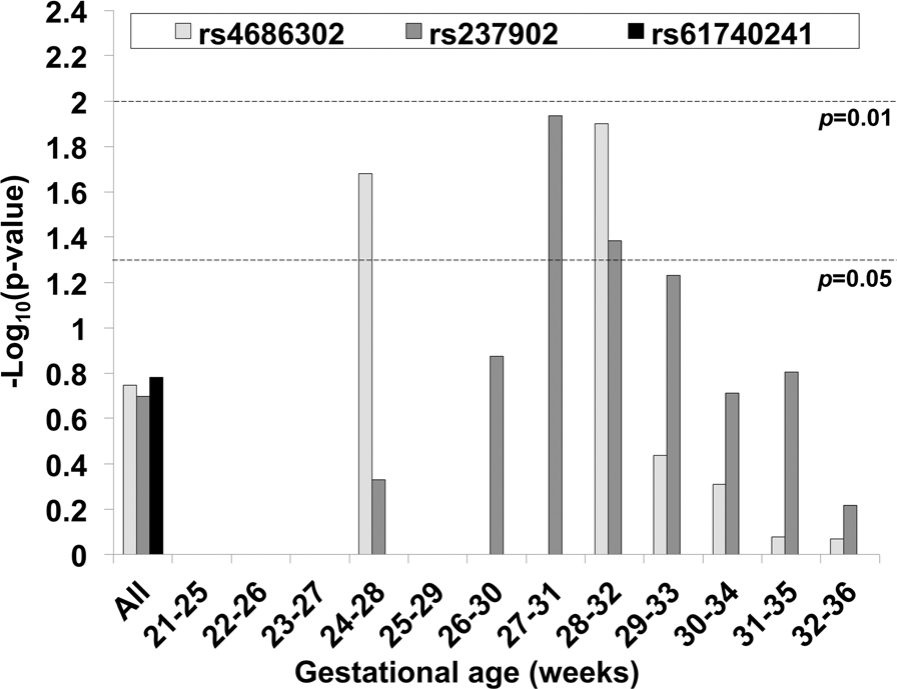
**Results of TDT analysis for three *OXTR *coding SNPs detected in U.S. maternal triads by sequencing.** Data on three *OXTR* SNPs (rs4686302, rs237902, and rs61740241) were analyzed on all GAs or five-week sliding window-stratified subgroups. Note that no data were available for some GA subgroups due to the lack of a sufficient number of informative families.

Among 8 coding variants identified by sequencing, two (Q143R and E242Q) were previously unreported novel variants (Table 3). Our TDT analysis did not reveal any significant association either when we examined all GAs together (Figure 4) or three subgroups (early, middle, and late) stratified by GA. (Of note, due to very low frequencies of rare variant minor alleles, complete results were available only for 3 SNPs (rs4686302, rs237902, and rs61740241) with more than 10 informative families.) However, when we performed a stratified analysis for the U.S. triads (*N*=109) using five-week sliding windows of GA, rs4686302 and rs237902 were found to be significant for the 24–28 and 28–32 week groups and the 27–31 and 28–32 week groups, respectively (Figure 4).

The three SNPs were further studied in the Iowa case–control study (71 case and 64 control mothers). We first developed logistic regression models to predict case/control status, and found that the model containing the two polymorphisms rs4686302 and rs237902 best explains case–control status (*p*=0.04, 0.05, respectively, R^2^=0.062) (Table 6). Next, linear regression analyses were performed to predict GA and BW. As demonstrated in Table 6, the models containing (rs237902 + rs61740241) and (rs4686302 + rs61740241) were best at predicting lower GA (*p*=0.03, 0.04, R^2^=0.055) and lower BW (*p*=0.008, 0.01, R^2^=0.074), respectively. The results indicate that 2 SNPs significantly explain 5.5% of the variation in GA and 7.3% of the variation in BW with rs61740241 in a slightly more supportive role to the other SNP. The SNPs that best predict case–control status are identical to the SNPs involved as the main predictors of GA in weeks and BW in grams.

**Table 6:** **Results of regression analyses of *OXTR *coding variants detected in Iowa case and control mothers**

**(A) Model**^**1**^	***n*(case, control)**	***P***		**R2**	
rs4686302 (A218T)	60, 60	0.19		0.020	
rs237902 (N230N)	60, 60	0.24		0.015	
rs61740241 (A238T)	60, 59	0.75		0.001	
rs4686302 (A218T) + rs237902 (N230N)^2^	60, 60	0.04, 0.05^3^		0.062	
**(B) Model**^**1**^	***n***	**Gestational age**		**Birth weight**	
		***P***	**R2**	***P***	**R2**
rs4686302 (A218T)	120	0.14	0.019	0.08	0.025
rs237902 (N230N)	120	0.23	0.022	0.17	0.016
rs61740241 (A238T)	119	0.77	0.001	0.53	0.003
rs237902 (N230N) + rs61740241 (A238T)^4^	120	0.03, 0.04	0.055	N/A	N/A
rs4686302 (A218T) + rs61740241 (A238T)^5^	120	N/A	N/A	0.008, 0.01	0.074

^1^(A) Logistic regression analysis to predict case/control status and (B) Linear regression analysis to predict gestational age and birth weight.

^2^The most parsimonious model for predicting case/control status.

^3^ rs4686302 OR=2.861 (1.041, 7.859) and rs237902 OR=1.803 (0.993, 3.273).

^4^The most parsimonious model for predicting gestational age.

^5^The most parsimonious model for predicting birth weight.

### Functional analysis of *OXTR* missense mutations and polymorphisms

**Figure 5:**
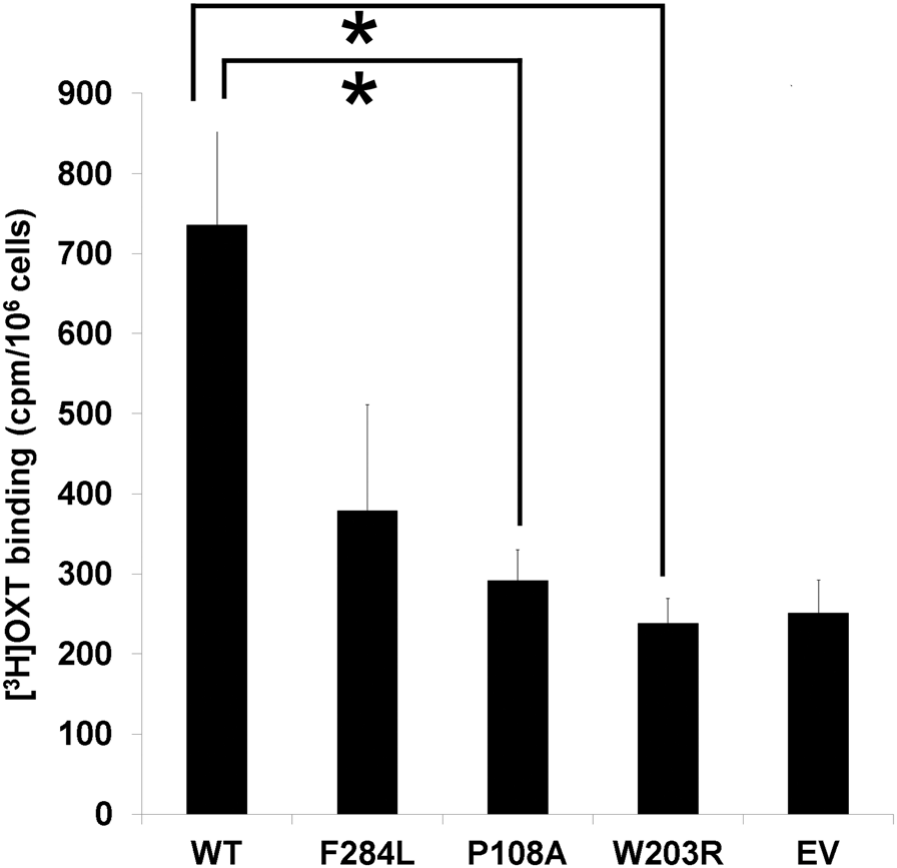
**Comparison of specific [**^**3 **^**H]OXT binding to wild-type and mutant oxytocin receptors.** COS-7 cells were transiently transfected with empty pcDNA3.1 vector and vectors encoding wild-type and mutant OXTRs, and were exposed to 50 nM [^3^H]OXT in the absence and presence of 20 μM unlabeled oxytocin. Specific binding was determined as described in Methods. Statistical significance of differences in [^3^H]OXT specific binding between wild-type- and mutant-transfected cells was determined using one-way ANOVA followed by Dunnett's test. Data presented are mean in cpm/10^6^ cells ± SEM of multiple experiments performed in duplicate. * *p*<0.05.

We performed *in vitro* functional studies on the aforementioned three *OXTR* missense mutations (P108A, W203R, and F284L) that were uniquely identified in our PTB cases and predicted as deleterious by PolyPhen [33] and SIFT [34] (Table 4). We first tested the ligand-binding properties of the mutant receptors, using transiently transfected COS-7 cells. To this end, the level of [^3^H]OXT binding was assessed in empty vector-, wild-type or mutant OXTR-expressing cells in the presence or absence of excess unlabeled OXT. The specific binding of [^3^H]OXT to intact transfected cells was defined as the difference between total and non-specific binding. We initially observed a considerable decrease in specific binding in cells transfected with the mutant plasmids, compared to those transfected with the wild-type plasmid. Because this could be a reflection of lower expression of the mutant receptor proteins, we attempted to increase their expression levels using twice more plasmid DNA (4 μg per well). However, the mean binding levels (in cpm/million cells) in each transfection group hardly varied. Based on this observation, we combined data from all experiments performed, regardless of the amount of transfected DNA. As shown in Figure 5, two of the three mutations (P108A and W203R) tested exhibited a statistically significant reduction in [^3^H]OXT binding. For the W203R mutation, there was nearly complete abolishment of specific binding. Our results suggest that these mutations may cause either reduced receptor expression or ligand affinity, leading to impaired ligand binding.

**Figure 6:**
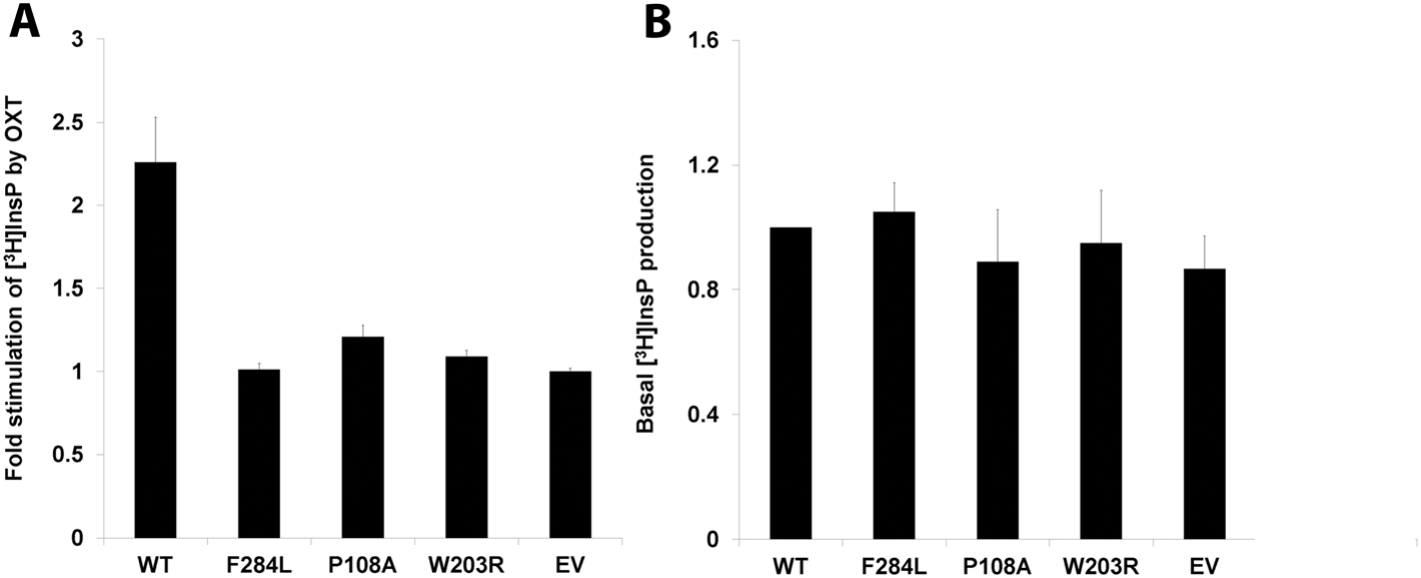
**Inositol phosphate (InsP) accumulation in COS-7 cells transiently transfected with wild-type and mutant OXTRs.** Transfected cells prelabeled with myo-[^3^H]inositol were treated with buffer alone or with 100 nM oxytocin. After one hour of incubation, inositol phosphate accumulation was measured as described in Methods. **(A)** Oxytocin-stimulated InsP accumulation. Results are presented as fold change compared with unstimulated (basal) InsP levels. **(B)** Comparison of basal levels of InsP accumulation in transfected cells. Results are presented as the ratio to the unstimulated (basal) InsP levels in cells expressing wild-type receptor. Data are mean ± SEM of multiple experiments performed in duplicate.

To gain insight into whether the missense mutations affect receptor signaling, OXT-stimulated inositol phosphate (InsP) production by wild-type and mutant OXTRs was evaluated. Figure 6A summarizes the results obtained. The accumulation of [^3^H]InsPs increased more than twofold in response to oxytocin in wild-type OXTR-expressing cells, while this increase was not observed in mutant plasmid- and empty vector-transfected cells. In addition, basal (unstimulated) levels of inositol phosphates were almost indistinguishable between wild-type OXTR and the three mutant receptors (Figure 6B), suggesting that the mutant receptors are unlikely to be constitutively active. Taken together, these data indicate that the examined mutations may have no effect on the coupling of the receptor to the second messenger signaling system.

## Discussion

Pregnancy and parturition involve an intricate biochemical and molecular interplay between mother and fetus [10]. The study of PTB has been hampered by incomplete knowledge of these complex, time-varying processes, and an investigation of static makers like sequence variants may serve as one way to predict the risk of PTB.

In this study, we used a candidate gene approach to better understand genetic factors contributing to PTB. Previous studies have identified some polymorphisms suggestively associated with PTB, but with a focus mostly on immune-related and inflammatory genes such as interleukin 1 receptor antagonist (*IL1RN*), interferon gamma (*IFNG*), and coagulation factor II (*F2*) [15]. The present study was undertaken to explore the contribution of genetic variations in another functionally relevant pathway, the oxytocin pathway to the etiology of PTB, given the important role of constituents of the pathway in labor and parturition.

Our analysis of fourteen SNPs in *OXT*, *OXTR*, and *LNPEP* revealed no evidence of fetal genetic effects on PTB. However, the analysis of maternal genetic effects performed all GA cases identified three SNPs in *LNPEP* that correlate with prematurity when not corrected for multiple comparisons. This finding is in line with the results of recent studies demonstrating that the maternal genome has a major impact on the risk of prematurity [35,36] and can be considered hypothesis generating for further investigations in the role of *LNPEP* in preterm labor (PTL).

Ishii *et al.*[37] reported that in LNPEP knockout mice, an increased sensitivity to OXT is observed and pregnancy duration is significantly shortened, providing functional evidence supporting our genetic data. It was noted that the same three *LNPEP* SNPs showing suggestive association in the analysis of all GAs remained significant in the GA-stratified analysis, further supporting the importance of these SNPs. The activity of LNPEP is known to gradually increase during late pregnancy and reach a very high level at 11 days before the onset of labor [37]. Interestingly, in our five-week sliding window analysis for maternal effects, the three SNPs were found to be significant in late GA groups (30–34, 31–35, and 32–36 week groups) although there was an additional significant SNP (rs13175726) detected for the 26–30 week group. Pending replication with larger samples, we postulate that these polymorphisms, all of which are non-coding SNPs, may serve as regulatory elements for the *LNPEP* gene rather than having a direct functional effect.

We screened the *OXTR* gene for rare genetic variation by resequencing and identified both previously reported and unreported rare variants in exon 3, which is highly conserved across species (Additional file 3). The variants identified were not found to be significantly associated with PTB, but these results should be interpreted cautiously because of the small sample size and the paucity of sequencing data on controls, especially those from Argentina and the U.S.

Our analysis comparing the rare variants identified by sequencing with those catalogued in the public databases demonstrates that some of the rare variants may be of etiologic importance. The observation that three (P108A, W203R, and F284L) of the four rare missense variants not found in either the 1000GP [28,29] or the NHLBI_ESP [30] data were predicted to affect the function of the receptor protein particularly merits further attention. These results suggest that rare *OXTR* coding variants appear to make some contribution to the genetic risk of prematurity, but larger studies will be needed to measure their combined as well as individual impact more accurately and determine the diagnostic or therapeutic relevance of the findings.

We studied the role of maternal *OXTR* genotype in PTB in more depth using maternal grandparent-mother triads. Transmission disequilibrium analysis revealed no significant transmission distortion of the *OXTR* coding variants examined when the analysis was performed on all GA cases or stratified into 3 GA groups. However, two missense SNPs (rs4686302 and rs237902) showed nominal associations in the U.S. triads for the sliding window GA subgroups primarily involving 24–32 weeks of gestation. These results suggest GA-specific effects of maternal genotypes of these *OXTR* SNPs on susceptibility to PTB.

We characterized the effects of three mutations of potential functional significance with *in vitro* assays. Our radioligand binding assay revealed that P108A and W203R mutant-transfected cells display significantly diminished levels of [^3^H]OXT specific binding, compared to wild-type-transfected cells. Although it should be further clarified if the mutations affect OXTR protein expression, ligand binding affinity, or both, our results indicate the importance of these residues in receptor function and support previous findings that the first and second extracellular loops of OXTR, where P108 and W203 residues, respectively, are located, are important for agonist binding and selectivity [38]. Despite their important biological effects, it is yet unclear how the mutations may contribute to the disease as it is counterintuitive that attenuated OXT signaling leads to PTB. However, it is possible that the variants may act indirectly to stimulate uterine contractions. Given that the regulation of OXTR function is dependent on steroids such as estradiol, progesterone, and its metabolites [39-41], it is probable that the mutations may affect receptor-hormone interaction in a way that interferes with the timely onset of labor.

Another mutation, F284L, also exhibited a reduced level of specific binding, but the result did not quite reach statistical significance. An earlier mutagenesis study has shown that when two transmembrane residues Y209 and F284 are mutated to F and Y residues, respectively, arginine vasopressin (AVP), a partial agonist of the OXTR, becomes a full agonist [8,42]. The finding indicates that these residues may be critical for modulation of the receptor response to AVP. Given the uterotonic action of AVP [43], an investigation into the sensitivity of the F284 as well as the other two mutants to other agonists like AVP could possibly provide an explanation about the pathogenic effects of those mutations.

Our study has certain limitations. First of all, the sample size of our association study may not be large enough to provide sufficient statistical power for the analysis of rare variants with small effects. Second, the functional assays were not carried out in a cellular context relevant to address mechanisms of PTB. The functional role of the identified *OXTR* mutations should be determined using physiologically more relevant cell types such as uterine myometrial cells. In addition, we only focused on the InsP pathway to examine the downstream effects of the *OXTR* mutations. We did not observe any significant difference in OXT-induced InsP production between wild type and mutant receptor-expressing cells, and it is possible that their effects are mediated through other signal transduction pathway(s). Therefore, it would be important to examine the impact of the mutations on different biologic pathways like the MAP kinase pathway [44].

## Conclusions

This study provides preliminary evidence for an association between maternal common polymorphisms in the *LNPEP* gene and the risk of PTB. In addition, we found that maternal *OXTR* missense SNPs rs4686302 and rs237902 may have gestational age-dependent effects on prematurity. These observations support that sequence variants in maternal genes may have a primary role in susceptibility to PTB. Most of the *OXTR* rare variants identified do not appear to significantly contribute to the risk of PTB. However, the importance of the non-synonymous rare variants shown to cause altered ligand binding warrants further investigation. Future genetic studies with larger sample sizes are needed to confirm the findings of the association analyses performed in the present study. Additional functional studies may provide more detailed insight into the precise physiological effects and clinical significance of the genetic variants identified and ultimately, accelerate translation of such findings into genetic-based diagnostic and therapeutic applications.

## Abbreviations

AVP: Arginine vasopressin; BW: Birth weight; CHR: Chromosome; dbSNP: Single nucleotide polymorphism database; DNBC: Danish National Birth Cohort; ECL: Extracellular loop; END: Extracellular N-terminal domain; FBAT: Family Based Association Test; F2: Coagulation factor II (thrombin); GA: Gestational age; HEPES: 4-(−2- hydroxyethyl)-1-piperazineethanesulfonic acid; HWE: Hardy-Weinberg equilibrium; ICL: Intracellular loop; IFNG: Interferon, gamma; InsP: Inositol phosphate; IL1RN: Interleukin 1 receptor antagonist; IRB: Institutional Review Board; LNPEP: Leucyl/cystinyl aminopeptidase; MAF: Minor allele frequency; MAP: Mitogen-activated protein; MT: Maternal triad; N/A: Not available; NHLBI_ESP: The National Heart, Lung, and Blood Institute Grand Opportunity Exome Sequencing Project; OR: Odds ratio; OXT: Oxytocin; OXTR: Oxytocin receptor; PLAP: Placental alkaline phosphatase; P1: Phase 1; P2: Phase 2; PTB: Preterm birth; PTD: Preterm delivery; PTL: Preterm labor; SIFT: Sorting intolerant from tolerant; SNP: Single nucleotide polymorphism; TDT: Transmission disequilibrium test; 1000GP: The 1000 Genomes Project; TMD: Transmembrane domain.

## Competing interests

The authors declare that they have no competing interests.

## Authors’ contributions

JK performed SNP genotyping, sequencing, comparative sequence analysis, and functional assays (including data analysis), and drafted and revised the manuscript and prepared figures and tables. KJS carried out SNP genotyping and sequencing, in part, and drafted part of the manuscript. MEC conducted statistical analyses of SNP genotype (fetal genetic effects) and sequencing (Danish cohort, maternal triads, and Iowa cases and controls) data, and assisted in study design. MA was involved in the analysis of functional assay data and assisted in study design. AMM participated in the management and preparation of genotyping and sequencing samples and data for analysis. KKR participated in statistical analysis of Danish sequencing data. ELM performed maternal triad sequencing. LR was involved in the management and sequencing of Danish cohort samples. KLS helped with sample organization. VC, EG, and CS coordinated patient recruitment, phenotyping, and sample collection, and assisted in study design. MS conducted statistical analysis of SNP genotype (maternal genetic effects) data. MH, JP, KAT, and LJM provided Finnish DNA samples. BF, FG, HAB, and MM provided Danish DNA samples and patient information, and were involved in study design and discussion of statistical analysis of Danish sequencing data. MLM participated in study design and discussion of statistical analysis of genotype and sequencing data. JMD assisted in study design and reviewed the draft of the manuscript. JCM is the principal investigator and obtained funding, established key collaborations, provided study design and supervision, and oversaw manuscript preparation. All authors read and approved the final manuscript.

## Pre-publication history

The pre-publication history for this paper can be accessed here:

http://www.biomedcentral.com/1471-2350/14/77/prepub

## Supplementary Material

Additional file 1: Table S1 List of rare missense variants in exon 3 of *OXTR* catalogued in public databases (1000GP, NHLBI_ESP). Click here for file

Additional file 2: Table S2 Results of regression analyses of *OXTR* coding variants predicting gestational age (GA) in Danish case and control mothers. Click here for file

Additional file 3: Table S3 Alignment of the amino acid sequence of *OXTR* from different species at mutant residues of all coding variants identified in the present study. Click here for file
