# Supplementary material for: Sequence variants in oxytocin pathway genes and preterm birth: a candidate gene association study
Source: BMC Med Genet. 2013 Jul 26;14:77. doi: 10.1186/1471-2350-14-77 (PMC3737028; doi:10.1186/1471-2350-14-77)
Supplement: Additional file 1: Table S1 — List of rare missense variants in exon 3 of OXTR catalogued in public databases (1000GP, NHLBI_ESP). [file 1471-2350-14-77-S1.pdf]

Additional file 1

Table S1 List of rare missense variants in exon 3 of *OXTR* catalogued in public databases (1000GP, NHLBI_ESP)

**dbSNP ID**	**Amino acid substitution**[1]	**1000GP**	**NHLBI_ESP**	
		**European**	**European American**	**African American**
		**mA / MA**	**mA / MA**	**mA / MA**
	**A11T**			
	**V45L**		7 / 8483	0 / 4348
	C47Y		0 / 8524	1 / 4365
rs143644523	L74F		1 / 8587	0 / 4394
rs202138705	**P108A**			
rs115324487	**V172A**	3 / 755	1 / 8599	243 / 4161
	W188C		1 / 8599	0 / 4406
	P194S		1 / 8599	0 / 4406
rs200498154	**W203R**			
rs150746704	**L206V**		1 / 8599	81 / 4325
	**V211L**			
rs143908202	G221S		0 / 8598	30 / 4374
	I227M		0 / 8598	1 / 4399
rs145921539	W228C		0 / 8596	6 / 4394
rs151141371	**G252A**		2 / 8590	0 / 4392
rs139854982	R254C		0 / 8594	1 / 4391
	V274I		1 / 8599	0 / 4406
rs144814761	**V281M**		4 / 8596	0 / 4406
rs201783860	**F284L**			

[1]Variants identified in the current study are shown in bold.

mA, minor allele count; MA, major allele count; 1000GP, the 1000 Genomes Project; NHLBI_ESP, the National Heart, Lung, and Blood Institute Exome Sequencing Project.
