# Supplementary material for: Sequence variants in oxytocin pathway genes and preterm birth: a candidate gene association study
Source: BMC Med Genet. 2013 Jul 26;14:77. doi: 10.1186/1471-2350-14-77 (PMC3737028; doi:10.1186/1471-2350-14-77)
Supplement: Additional file 2: Table S2 — Results of regression analyses of OXTR coding variants predicting gestational age (GA) in Danish case and control mothers. [file 1471-2350-14-77-S2.pdf]

Additional file 2

Table S2 Results of regression analyses of *OXTR* coding variants predicting gestational age (GA) in Danish case and control mothers[1]

Variant	GA in weeks[2]		GA as a binary trait[3]	
	n[4]	*P*	*n* (case, control)[4]	*P*
rs237913[5]	1591	0.08	691, 900	0.11
rs237911	1598	0.22	696, 902	0.44
A11T	1602	0.53	697, 905	0.98
V45L	1602	0.28	696, 906	0.97
V211L	1590	0.39	698, 892	0.86
rs4686302 (A218T)	1592	0.57	701, 891	0.54
rs237902 (N230N)	1586	0.26	697, 889	0.35
rs61740241 (A238T)	1588	0.46	698, 890	0.26
A247A	1587	0.79	696, 891	0.44
rs151141371 (G252A)	1588	0.35	698, 890	0.25
rs144814761 (V281M)	1588	0.38	697, 891	0.98

[1]The analysis was performed for all common and rare variants identified by sequencing.

[2]GA was considered as a continuous variable.

[3]GA was considered as a categorical variable (preterm, ≤36 weeks; and term, 40 weeks).

[4]Number of individuals successfully genotyped and included in the analysis.

[5]The best genetic effect model.
