# Supplementary material for: Sequence variants in oxytocin pathway genes and preterm birth: a candidate gene association study
Source: BMC Med Genet. 2013 Jul 26;14:77. doi: 10.1186/1471-2350-14-77 (PMC3737028; doi:10.1186/1471-2350-14-77)
Supplement: Additional file 3: Table S3 — Alignment of the amino acid sequence of OXTR from different species at mutant residues of all coding variants identified in the present study. [file 1471-2350-14-77-S3.pdf]

**Additional file 3**

**Table S3 Alignment of the amino acid sequence of *OXTR* from different species at mutant residues of all coding variants identified in the present study**

| Amino acid residue | Human | Rhesus | Mouse | Dog | Elephant | Opossum | Chicken | X_tropicalis | Zebrafish |
|---|---|---|---|---|---|---|---|---|---|
| **A11** | A | T | I | A | A | L | — | — | — |
| **V45** | V | V | V | V | V | V | V | V | V |
| **P108** | P | P | P | P | P | P | P | P | P |
| **Q143** | Q | Q | Q | Q | Q | Q | Q | Q | Q |
| **V172** | V | V | V | V | V | I | I | I | V |
| **W203** | W | W | W | W | W | W | W | W | W |
| **L206** | L | L | L | L | L | L | L | I | L |
| **V211** | V | V | V | V | V | I | I | L | I |
| **A218** | A | A | A | A | A | A | V | V | V |
| **N230** | N | N | N | N | N | N | N | N | N |
| **A238** | A | A | A | A | A | E | G | V | E |
| **E242** | E | E | A | E | E | A | G | — | — |
| **A247** | A | A | A | A | E | S | S | E | L |
| **G252** | G | G | A | — | G | G | G | R | S |
| **V281** | V | V | V | V | V | V | V | V | V |
| **F284** | F | F | F | F | F | F | F | Y | Y |
